# Protective effects of grape seed and skin extract against high-fat-diet-induced lipotoxicity in rat lung

**DOI:** 10.1186/s12944-017-0561-z

**Published:** 2017-09-13

**Authors:** Mohamed El Ayed, Safwen Kadri, Selima Smine, Salem Elkahoui, Ferid Limam, Ezzedine Aouani

**Affiliations:** 1 Bioactive Substances Laboratory, Biotechnology Centre, Technopolis Borj-Cedria, BP-901, 2050 Hammam-Lif, Tunisia; 2 Proteomic Platform PISSARO, Institut de Recherche et d’Innovation Biomédicale (IRIB), University of Rouen, 76821 Mont Saint Aignan, Cedex France

**Keywords:** Obesity, Asthma, Grape seed extract, Fatty acids

## Abstract

**Background:**

Obesity is a public health problem characterized by increased fat accumulation in different tissues. Obesity is directly linked to breathing problems and medical complications with lung, including obstructive sleep apnea syndrome, obesity hypoventilation syndrome, chronic obstructive pulmonary disease, asthma….In the present work, we aimed to investigate the effect of high fat diet (HFD) on lung lipotoxicity, oxidative stress, fatty acid composition and proportions in lung and implication in asthma development. The likely protection provided by grape seed extract (GSSE) was also investigated.

**Methods:**

In order to assess HFD effect on lung and GSSE protection we used a rat model. We analyzed the lipid plasma profile, lung peroxidation and antioxidant activities (SOD, CAT and POD). We also analyzed transition metals (Ca2+, Mg2+, Zn2+ and iron) and lung free fatty acids using gas chromatography coupled to mass spectrometry (GC-MS).

**Results:**

HFD induced lipid profile imbalance increasing cholesterol and VLDL-C. HFD also induced an oxidative stress assessed by elevated MDA level and the drop of antioxidant activities such as SOD, CAT and POD. Moreover, HFD induced mineral disturbances by decreasing magnesium level and increasing Calcium and iron levels. HFD induced also disturbances in lung fatty acid composition by increasing oleic, stearic and arachidonic acids. Interestingly, GSSE alleviated all these deleterious effects of HFD treatment.

**Conclusion:**

As a whole, GSSE had a significant preventive effect against HFD-induced obesity, and hence may be used as an anti-obesity agent, and a benefic agent with potential applications against damages in lung tissue.

## Background

Obesity is a chronic disease characterized by excessive accumulation and storage of body fat that is harmful to individuals. Over 1.9 billion adults worldwide are overweight, of which 600 million are obese [1]. The prevalence and incidence of obesity has significantly increased during the past 30 years in the world. Obesity is a multifactorial disease which depend on the interaction of multiple factors including genotype, environment, lifestyle and the unbalance between diet and caloric requirements.

The increased morbidity and mortality associated with obesity is reflected by numerous chronic diseases, including cardiovascular and metabolic diseases [2], hypercoagulable states [3], lower back pain [4], osteoarthritis [5], and cancer [6].

Possible consequences of obesity on respiratory system include a restrictive disorder, changes in ventilatory mechanics and an alteration of respiratory drive.

Obesity has been linked to lung problems and respiratory symptoms. Obesity around the abdomen further leads to worsening lung function. It is hypothesize that accumulation of fat tissue in the abdominal wall and around the abdominal organs hampers movement of the diaphragm, reduce the lung expansion during inspiration, and reduced lung capacity. Moreover, lipid accumulation on the anterior chest wall lowers chest wall compliance and respiratory muscle endurance and increases airway resistance. However, the biological basis for the relationship between asthma and obesity has not been established but several mechanisms has been proposed [7].

Grape seed extract (GSSE) is a natural product which contain several active components including flavonoids, polyphenols, proanthocyanidins, procyanidines, anthocyanins and the stilbene derivative resveratrol [8].

It has been reported that GSSE possesses a broad spectrum of pharmacological and therapeutic effects such as anti-inflammatory [9], antioxidative (Dogan et Celik 2012), antiaging [10], anticarcinogenic [11], as well as having hepatoprotective [12], cardioprotective [13], and neuroprotective effects [14]. All these pharmacological effects made the GSSE a potential treatment for obesity-induced asthma.

The aim of the present study is to investigate on HFD-induced lung toxicity and oxidative stress and to determine physiological connections between HFD and asthma. We further aimed to investigate the protective effect to GSSE on HFD-induced lung disturbances.

## Methods

### Reagents and diets

Grape seed and skin extract (GSSE) was processed from a grape cultivar (Carignan) of *Vitis vinifera* from northern Tunisia. Polyphenolic powder mixture containing grape seed (50%) and skin (50%) was dissolved in 10% ethanol (*v*/v). After rigorous shaking and centrifugation (10,000 g 15 min 4 °C), supernatant containing soluble polyphenols was used daily and administered by intraperitoneal injection (i.p). High-fat diet (HFD) was prepared by soaking commercial food pellets into warmed (100 °C) and liquefied fat (peri-renal) from animal origin (sheep) during 15 min and allowed to dry at room temperature.

### Animals treatment and lung tissue sample preparation

Twenty-four male Wistar rats (210–230 g) were used in this study, in conformity with the NIH guidelines (National Research council, 1985). They were maintained in animal facility at controlled temperature (22 ± 2 °C), a 12 h light/dark cycle, and divided into four groups of six animals each fed either a standard diet (SD) or a HFD for 6 weeks. The HFD consisted of 40% fat, 45% carbohydrate, and 15% protein. The SD consisted of 5%fat, 70% carbohydrate, and 25%protein (ALMAS, Bizerte, Tunisia).

HFD contained essentially fat from animal origin (sheep) whose composition is given in Table 1. Food intake is defined as the amount of food consumed in g per 24 h.

**Table 1:** Fatty acid composition of sheep fat (September 2016)

Fatty acid	Relative abondunce (%)	Class
C14: 0	0,17	Saturated fatty acid 71,7%
C16: 0	24,69	
C18: 0	46,74	
C20: 0	0,09	
C22: 0	0,01	
C14: 1n5	0	Monounsaturated fatty acid 26,62%
C16: 1n7	0,2	
C18: 1n9	26,3	
C20: 1n9	0,12	
C18: 2n6	1,12	Polyunsaturated fatty acid 1,69%
C18: 3n3	0,08	
C20: 2n6	0,01	
C20: 5n3	0,04	
C22: 5n3	0,42	
C22: 6n3	0,02	

Rats additionally received by daily i.p injection either 10% ethanol as vehicle (control SD and HFD) or 500 mg/kg bw GSSE (SD + GSSE and HFD + GSSE). At the end of the treatment period, rats were sacrificed by decapitation; their blood collected using heparin as anticoagulant and plasma processed for the determination of lung function indicators and free fatty acids (FFAs).

Lung tissue was carefully dissected, weighed, and homogenized in phosphate buffer saline pH 7.4 with an ultrathurax T25 homogenizator. Homogenates were centrifuged at 10000 g for 15 min at 4 °C.

### Lung lipidemia

Lung lipids were extracted according to Folch et al. (1957). Triglyceridel was determined by using a commercial kit from Biolabo SA (France).

Lipase activity (E.C. 3.1.1.3) was determinated according to Humbert et al. using P-nitrophenol dodecanoate as substrate.

### Plasmatic assays

The lipid profile includes total cholesterol (TC), triglycerides (TG), low-density lipoprotein cholesterol (LDL), very low density lipoprotein cholesterol (VLDL) and high-density lipoprotein cholesterol (HDL). The serum levels lipid profile was determined by using a commercial kit from Biolabo SA (France).

C reactive protein (CRP) was measured using a Konelab clinical chemestry analyser (Thermoclinical, Labsystems, Finland).

Glucose was determined using commercially available kits from Biomagreb (Tunisia).

Adiponectin was measured using the Assay Max rat adeponectin ELISA Kit (Assay paro) and plasma insulin was measured using the ultrasensitive rat insulin ELISA Kit (Alpco Diagnostics).

### Lipoperoxidation

Malondialdehyde (MDA), a marker of lipid peroxidation was determined according to Draper and Hadley (1990). An aliquot of the homogenate was mixed with butylated hydroxytoluene/trichloro-acetic acid (BHT/TCA) solution containing 1% (*w*/*v*) BHT dissolved in 20% TCA (w/v) and centrifuged at 4000 g for 15 min at 4 °C. Supernatant was blended with 0.6 N HCl and 120 mmol·L − 1 thiobarbituric acid in a 26 mmol·L − 1 Tris buffer. The mixture was heated at 80 °C for 10 min, cooled, and absorbance measured at 532 nm. MDA concentration was calculated using the absorbance coefficient MDA-TBA complex 1.5610 5 cm-1 M-1.

### Anti-oxidant enzyme activities

Lung homogenates were also used for endogenous antioxidant enzyme activities as peroxidase (POD; E.C.1.11.1.9.) according to Nakamura et al. (1974)., catalase (CAT; E.C.1.11.1.6.) according to the method of Aebi (1974), and superoxide dismutase (SOD; E.C.1.15.1.1.) according to Misra and Fridovich (1972). This method is based on the capacity of SOD to inhibit autoxidation of adrenaline to adrenochrome.

### LDH activity measurement

Lactate dehydrogenase (LDH) activity (E.C.1.1.1.27) was assayed spectrophotometrically according to [15] using a commercial kit from Bio-Maghreb Tunisia. Briefly in the presence of pyruvate and NADH/H+, LDH produces lactate and NAD +. LDH activity was measured following the decrease in NADH at 340 nm.

### Transition metals levels

Calcium, iron, zinc and magnesium were assessed using an ICP-OES analysis on six rats from each group. Briefly, lungs were chemically hydrolyzed with nitric acid (15.5 mol L-1) diluted, and filtered. Zinc and magnesium in the mineralized samples were analyzed with a dual view ICPOES.

### Mass spectrometry analysis of free fatty acids

#### Sample preparation

Triplicate subsamples of 10 mg were extracted according to Folch et al. A chloroform/methanol (Labscan Ltd) mixture (2:1 *v*/v) was used for total lipid extraction. After washing with water and centrifugation at 8000 rpm for 10 min, the organic layer containing total lipid was recovered and dried under a stream of nitrogen. The residue was dissolved in a known volume of toluene/ethanol (4:1 *v*/v) at −20 °C for further analysis. Total lipid extraction was performed in triplicate.

#### GC-MS analysis of fatty acid methylation

Total fatty acids were converted into their methyl esters using 20 gL-1 sodium methylate in methanol according to the method described by [16]. Free fatty acid methyl esters were analyzed using a Agilent GC–MS system (GC with 7890A, mass detector 5975C with Triple-Axis, insert XL MSD). The temperature of the oven was programmed at 70 °C for 2 min, raised to 230 °C for 20 min and raised again to 270 °C for 25 min. The MS used in this analysis had the following characteristics: source and transfer line temperature at 250 °C, ms quadrupole at 150 °C, ion ionized energy 70 eV. The scan time and mass range were 1 s and m/z 50 to 550 respectively. The identification of the fatty acid were done with the wiley 09 NIST2011 library. The percentage determination was based on peak area normalization without using correction factors.

### Statistical analysis

All Data were subjected to statistical analysis using Statistica software (Tulsa, okla, USA). Statistical differences for each group were evaluated by unpaired one-way analysis of variance (ANOVA). Results are expressed as means ± S.E.M and a *p* value less than 0.05 was considered statistically significant.

## Results

### Grape seed and skin extract composition

Analysis of different polyphenolic classes (Table 2) showed that grape seed and skin contains respectively 67 and 51 mg/g GSSE of total polyphenols, 16 and 14 mg/g GSSE of total flavonoids, 51 and 37 mg/g GSSE of non-flavonoids and 1,22 and 3,43 mg/g GSSE of condensed tannins. Chromatographic compounds analysis showed that the major polyphenols in the grape and skin are Galic acid with 50.3 and 32.77%; 2,5-dihydroxybenzoïc acid with 30.58 and 51.96% respectively, followed by vanillin with 10.67 and 7.75% (Table 3).

**Table 2:** Polyphenols content in the Grape seed and grape skin (September 2016)

Polyphenols	Grape seed	Grape skin
Total polyphenols (mg/g GSSE)	67	51
Total flavonoids (mg/g GSSE)	16	14
non-flavonoids (mg/g GSSE)	51	37
Tannins (mg/g GSSE)	1.22	3.43
Anthocyanins (μg/g GSSE)	0.997	0.962

**Table 3:** GSSE polyphenols (LC–MS/MS) (September 2016)

Compounds	m/z negative mode [M-H]^−^	MS^2^ fragment	Abondance relative (%)	
			Pépins	Peau
Catechin	289	245/108.8/122.8	2.27	0.36
Epicatechin	289	245/108.8/122.8	2.85	0.37
Procyanidin dimmer	577	289.3/407.4	0.47	ND
Procyanidin trimer	865	577	ND	ND
Quercetin	301	150.8/120.9	0.64	0.47
Resveratrol	227	184.6/143	0.14	ND
Rutin	609.19	300.1	1.51	0.5
Vanillin	151.14	135.7/108.1	10.67	7.75
Gallic acid	169	124.7/78.9	50.3	32.77
P-coumaric acid	163	119/93	ND	0.38
Rosmarinic acid	359.2	160.8/197.1	ND	0.75
2,5-dihydroxybenzoïc acid	152.7	108.7/90.7	30.58	51.96
Caffeic acid	179	135	ND	2.8
Chlorogenic acid	353	191	ND	0.34
Ferulic acid	193	134/89	0.55	1.46

### Effect of HFD on biometric parameters body weight, lung weight and lung lipid content

High fat diet also affected biometric parameters (Table 4) and increased plasma CRP by 175%. However, high fat diet did not affect plasmatic levels of insulin or glucose and decrease plasma adiponectine by 44%. Moreover, After 60 day of high fat diet, body weight of HFD group reached 317,2 g with 33,89% of overweight compared to SD rats. GSSE prevented weight gain and maintained rats weight near to SD levels. HFD group lung weight also increased significantly with 25% compared to SD group, whereas HG group maintained lung weight in normal levels compared to SD group. Lung lipid content also increased with 31% in HFD group compared to SD group. GSSE treated groups have less lipid content compared to SD group (Table 5).

**Table 4:** Effect of HFD and GSSE on biometric parameters (December 2016)

	SD	HFD	SG	HG
Plasma CRP (mg/l)	0.20 ± 0.05	0.55 ± 0.05^a^	0.11 ± 0.01	0.35 ± 0.06^a, b^
Glucose (mg/dl)	102 ± 0.25	115 ± 0.65	105 ± 0.45	109 ± 0.55
Plasma insulin (ng/mL)	1.75 ± 0.03	1.55 ± 0.01	1.95 ± 0.01	1.67 ± 0.01
Plasma adiponectin (ng/mL)	6.20 ± 0.02	2.75 ± 0.04^a^	6.30 ± 0.01	4.95 ± 0.02^a, b^

^a^significant compared to SD

^b^significant compared to HFD

**Table 5:** Effect of high-fat diet (HFD) and grape seed extract on body weight, lung Index and lipid content. (December 2016)

	SD	HFD	SG	HG
Body weight	237,8 ± 48,83	317,2 ± 16,63^a^	228,2 ± 14,77	246,4 ± 10,07^b^
Lung weight	1,526 ± 0,05095	1,916 ± 0,0332^a^	1,604 ± 0,08226	1,648 ± 0,08823^b^
Lung lipid content	0,1467 ± 0,0145	0,1933 ± 0,0088^a^	0,1100 ± 0,0057	0,1233 ± 0,0145^b^

^a^significant compared to SD

^b^significant compared to HFD

### Effect of HFD on lipid profile and biometric parameters

We further sought to determine the effect of high-fat-diet on lipid profile (Table 6). High-fat diet clearly elevated triglyceride with 25%, cholesterol with 46%, HDL cholesterol 90%, LDL cholesterol 50% and HTR with 30%. GSSE treatment successfully backed triglyceride, cholesterol, LDL cholesterol and HTR to near control level, while HDL cholesterol level was maintained higher to control level with 13% elevation. HFD treatment was not diabetogenic as it did not affect insulinemia and glycemia but was obesogenic as indicated by decreased adiponectinemia.

**Table 6:** Effect of high-fat diet and GSSE on lipid profile (December 2016)

	SD	HFD	SG	HG
Triglyceride	0,15945 ± 0,0169	0,54265 ± 0,0207^a^	0,10385 ± 0,0134^a^	0,1934 ± 0,01^b^
Cholesterol	1,46495 ± 0,0174	4,08125 ± 0,0143^a^	1,03285 ± 0,0104^a^	1,7365 ± 0,0208^b^
HDL	0,3488 ± 0,0033	0,2827 ± 0,0072^a^	0,3059 ± 0,0023^a^	0,3827 ± 0,0023^b^
LDL	0,3189 ± 0,0121	1,0853 ± 0,0080^a^	0,2077 ± 0,0045^a^	0,3868 ± 0,0017^b^
LDL/HDL	0,91427 ± 0,0188	3,8390 ± 0,0208^a^	0,67898 ± 0,0132^a^	1,01071 ± 0,0205^b^
VLDL	0,03189 ± 0,0038	0,10853 ± 0,0078^a^	0,02077 ± 0,0019^a^	0,03868 ± 0,0020^b^
HTR	0,2380 ± 0,0239	0,0692 ± 0,0065^a^	0,2961 ± 0,0808	0,2203 ± 0,0469^b^
AI	3,199 ± 0,201	13,43 ± 1,240^a^	2,376 ± 0,187^a^	3,537 ± 0,418^b^

^a^significant compared to SD

^b^significant compared to HFD

### Effect of HFD on antioxidant activity and H_2_O_2_

**Fig. 1:**
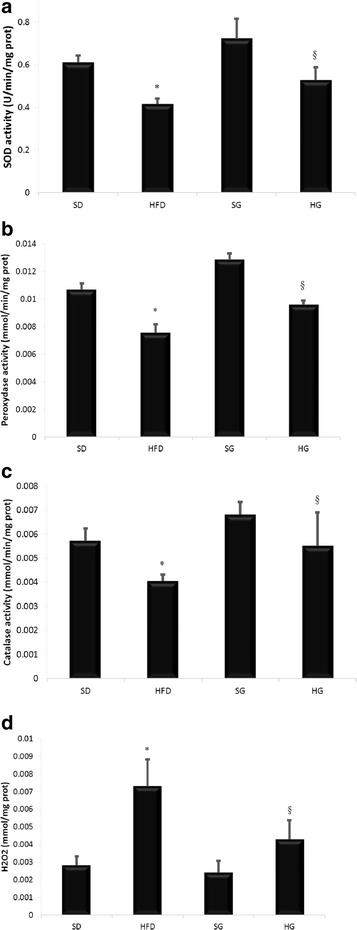
GSSE protective effect on high-fat diet-induced decrease of antioxidant activity and H_2_O_2_ increase; **a** superoxide dismutase, **b** Peroxidase activity, **c** Catalase activity, **d** H_2_O_2_ (December 2016)

We further thought to determine the effect high-fat-diet on antioxidant activity and hydrogen peroxide levels (Fig. 1). High-fat-diet decreased all antioxidant activities, it decreased SOD activity by 33%, Peroxidase activity by 27% and Catalase activity by 30%. High-fat-diet provoked also hydrogen peroxide increasing by 150%. GSSE treatment backed them to near control levels.

### Effect of HFD on lactate dehydrogenase

**Fig. 2:**
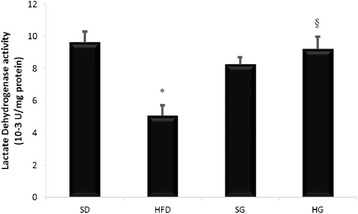
GSSE protective effect on high-fat diet-induced decrease of lactate dehydrogenase activity (December 2016)

Lactate dehydrogenase activity was modified under HFD condition. In fact, HFD induced the decrease of lactate dehydrogenase activity by 50% whether GSSE prevented this drop and maintained lactate dehydrogenase activity near to the control level (Fig. 2).

### Effect of HFD on transition metals

**Fig. 3:**
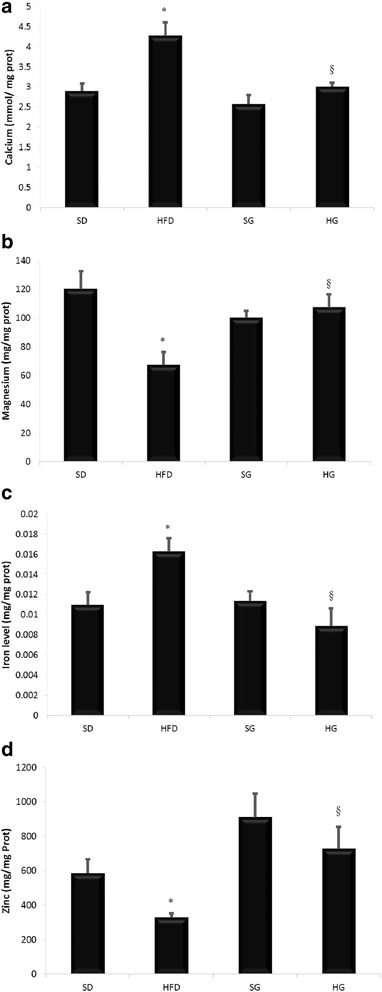
GSSE protective effect on high-fat diet-induced transition metals disturbance **a** Calcium **b** Magnesium **c** Iron **d** Zinc (January 2017)

We further asked if the high-fat diet affected the homeostasis of lung transition metals (Fig. 3). High-fat-diet decreased calcium, magnesium and zinc by almost 50% both and increased free iron by 45%. GSSE prevented all these disturbances and maintained transition metals to near control levels.

### Effect of HFD on lung lipid peroxidation and lipase activity

**Fig. 4:**
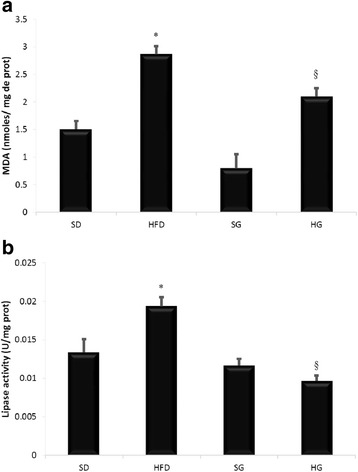
Protective effect of GSSE on high-fat diet-induced lipid peroxidation and lipase activity disturbance **a** lipoperoxidation **b** lipase activity (December January 2016/2017)

High-fat-diet altered lipid homeostasis and provoked the increase of lipid peroxidation with almost 90% rise compared to standard diet level. High-fat-diet affected also lipase activity which increased with 50%. GSSE treatment restores lipid peroxidation and lipase activity to near control level (Fig. 4).

### Effect of HFD on lung fatty acid composition

Fatty acid composition was disturbed with hat-fat-diet (Table 7). Myristic acid (C14:0) increased in SG, HFD and HG groups. Palmitic acid (C16:0) and Stearic acid (C18:0) was constant in all groups. Palmitoleic acid (C16:1n9) decreased in SG, HFD and HG groups. Oleic acid (C18:1n9) increased in HFD group. Vaccenic acid (C18:1n11) increased in HFD group. Linoleic acid (C18:2n9) decreased in HFD group. Arachidonic acid (C20:4n6) decreased extremely in HFD group and increased in HG goup. Saturated fatty acids increased in SG, HFD and HG groups while monounsaturated decreased in SG and HG groups. Polyinsaturated fatty acids decreased in HFD groups and oppositely decreased in HG group.

**Table 7:** Effect of high-fat diet and GSSE on lung fatty acid composition (January 2017)

Fatty acid	Relative abundance (%)			
	SD	HFD	SG	HG
C14: 0	2,59 ± 0,62	0,9 ± 0,6^a^	3,73 ± 0,71	3,83 ± 0,73^b^
C16: 0	41,31 ± 2,42	36,1 ± 1,97^a^	40,7 ± 1,57	41,11 ± 1,21^b^
C18: 0	8,33 ± 0,84	10,31 ± 0,95^a^	8,06 ± 1,07	8,58 ± 0,56^b^
Saturated	52,23 ± 2,17	47,31 ± 1,54^a^	52,49 ± 0,94	53,52 ± 1,01^b^
C16: 1n9	7,5 ± 1,07	8,42 ± 1,24	7,71 ± 0,89	5,42 ± 1,42^a^
C18: 1n7	29,06 ± 1,52	29,23 ± 0,91	29,47 ± 1,71	30,65 ± 2,09
C18: 1n9	1,57 ± 0,27	3,54 ± 0,84^a^	1,34 ± 0,17	1,16 ± 0,57
Monoinsaturated	38,13 ± 1,14	41,19 ± 0,93^a^	38,52 ± 1,01	37,23 ± 1,58
C18: 2n6	6,41 ± 0,88	5,47 ± 1,20	5,44 ± 0,57	5,98 ± 0,79
C20: 4n5	3,23 ± 0,61	6,03 ± 0,96^a^	3,55 ± 0,82	3,27 ± 0,49
Polyinsaturated	9,64 ± 0,61	11,5 ± 1,18^a^	8,99 ± 0,79	9,25 ± 0,68

^a^significant compared to SD

^b^significant compared to HFD

## Discussion

High-fat-diet rodent model is widely used to study human obesity. It’s a model that mimics human obesity and its-related disturbances. Two months High-fat-diet lead to an overt obesity in rats characterized by body weight gain, along with an increase of lung weight. Excessive ingestion of lipids, through a relatively long period, induced dyslipidemia into plasma as assessed by high cholesterol and LDL-C and high LDL-C/HDL-C ratio and into lung by high triglyceride level and high lipid content. HFD provokes lipotoxicity without affecting insulinemia nor glycemia and decreases adiponectinemia. Our data rather indicated the lipotoxic but not glucotoxic effect of this specific HFD. Lipid deposition in lung induced oxidative stress through the increase of free radical levels as evidenced by hydrogen peroxide elevation, lipid peroxidation augmentation, and the decrease of antioxidant enzymes activities such as SOD, CAT and POD. Similar HFD-induced oxidative stress was described by many previous studies. However, high-fat diet-induced oxidative stress mechanism is not well elucidated. Several reports proposed that obesity-induced oxidative stress is provoked by exacerbated nutrient oxidation, such as beta-oxidation, as it has been reported after glucose uptake [17]. We do think that high-fat diet-induced oxidative stress may by induced from excessive mitochondria functioning which can cause mitochondria damages and by decreasing peroxisome proliferator-activated receptor γ (PPAR- γ) due to fatty acid accumulation [18]. PPAR- γ downregulation decrease carnitine palmitoyl transferase-1 expression and fatty acid-oxidation. High-fat diet also affected antioxidant enzymes activities such as SOD, CAT and POD. These results are in accordance with previous works [19] although CAT variation seems to by organ specific.

HFD treatment also affected metals ions homeostasis. We found that HFD induced the depletion of magnesium and the accumulation of calcium and free iron within lung. Similar results were obtained previously showing that 24 week high-fat diet induces magnesium decrease in heart of DBA/2 mice [20] and calcium increase in brain [21]. However, HFD effect on calcium homeostasis is tissue specific. In fact, HFD induces calcium increase in the brain and oppositely its decrease in the heart [20, 21]. We might think that magnesium depletion are strictly related to free iron accumulation. In fact, iron accumulation will provoke cell depolarization and the enhance of divalent metal ion export resulting in other ions depletion such as Cu^2+^, Zn^2+^, and Mn^2+^ as assessed by zinc depletion [20, 22]. LDH decrease is most probably related to the decrease in lung zinc which will consequently affect aerobic/anaerobic switch in lung cells. Several studies assessed LDH activity increase in bronchoalveolar lavage fluid or in plasma [23, 24] but no studies have evaluated the LDH activity within lung cells. Moreover, HFD reduced lung lipase activity which is in accordance with triglyceride accumulation and the increase of lung lipid content and lung weight. Previous works support our data [25] and demonstrated the link between iron status and lipid metabolism, in particular the ability of free iron to inhibit lipoprotein lipase activity and consequently hypertriglyceridemia.

Calcium and magnesium are very important in lung function. Calcium play a key role in the shorting and contraction of airway smooth muscles [26]. Although, HFD effect on calcium homeostasis is tissue specific, HFD inducing-calcium metabolism disturbances pathway remain unclear. One possible way is that elevated free radicals level can lead to increased intracellular calcium through the activation of PLC by the H2O2 [27]. Other possible way, is the increase of GPR 40 activation which will increase calcium efflux from RE through PLC-IP3 pathway [28]. On the other side HFD-triggered magnesium depletion form lung. Magnesium is very important in lung function. In fact, magnesium plays a crucial role in the regulation of bronchial smooth muscle contractility and hyper-responsiveness [29]. Magnesium depletion was associated with several diseases such as type 2 diabetes and metabolic syndrome [30].

Lung is the organ which contain the biggest part of body fatty acids with 85% [31]. Analysis of fatty acid lung composition showed that oleic acid (C18:1n9) and arachidonic acid (C20:4n5) increased in HFD group. Oleic acid represent a large portion of ingested fatty acids in HFD. It is present in human plasma and cell membrane [32]. It has been demonstrated that high oleic acid levels can promote lung injury and inflammation. In fact, after intravenous inoculation, oleic acid, targeted lung and provoked lung lesion [33], neutrophil accumulation [34] and inflammatory mediators increase such as TNF훼 and IL-8 [35]. Since, major HFD fatty acids are stearic and oleic acid, we do think that major HFD-induced disturbances are mediated from these two fatty acids. In fact, stearic acid suppresses T cells activity leading to the loss of inflammation control. HFD also induced the increase of arachidonic acid. Arachidonic acid is either imported from nutrition or produced form linoleic acid throw the activity of fatty acid desaturase and fatty acid elongase. It can be produced also from diacylglycerol or phospholipids throw the activation of phospholipase A2 and phospholipase C. Arachidonic acid is a precursor of prostaglandin E_2_ and F_2α_ which plays an important role in inflammation. Concomitant effect of stearic and oleic acids along with high arachidonic acid level will result in chronic inflammation which can promotes asthma.

Many studies described the putative effect of dietary on asthma and lung diseases and specially fatty acids rich dietary. Here we found evidence of the linkage between fatty acids disturbances and asthma disturbances. In fact, disturbances induced by HFD on calcium magnesium, and free iron homeostasis are similar to asthma dysregulation. Recently, free iron accumulation into lung was associated with asthma and lung dysfunction [36]. Similarly, calcium increases and magnesium decreases in asthmatic lung [37]. Same disturbances are found in HFD treated group. HFD implement a global status which promotes asthma throw the activation of inflammation (by the oleic, stearic and arachidonic acids), the increase of oxidative stress and the disturbances of metal ions. All these imbalances provide the optimal physiological condition for asthma development.

Interestingly, GSSE prevented all major HFD-induced disturbances namely calcium increase, magnesium decrease and fatty acids imbalance. GSSE exert many protective effects on different diseases such as brain ischemia [14], hypertension [38].... It has been also showed that GSSE protect the organism from many HFD-induced damages such as brain lipotoxicity [21], liver and heart oxidative damages [39]. GSSE conferred protection is probably due to its effect on one or different levels of fattening process. In fact, GSSE likely affect intestinal absorption and blood transport of lipids as evidenced by blood lipid profile and body weight. Recent study showed that GSSE inhibit digestive lipase [40] and modulate intestinal absorption of lipids throw the regulation intestinal Fxr-target gene expression [41]. Additionally, GSSE affectes tissue lipid storage as evidenced by lung lipid content and triglyceride level. Similar effect of GSSE on triglyceride accumulation where previously observed [42]. Other putative effect of GSSE is to inhibit lipid entering into cells and decreasing receptors sensibility to fatty acids. There is no evidence of the possible interaction between GSSE and GPR receptors, thus, further investigation are needed.

GSSE has also an effect on antioxidant activities by increasing POD activity. GSSE effect on antioxidant activities was previously reported in many organs such as heart [43] and brain [14]. Furthermore, GSSE protected lung from HFD-induced oxidative stress preventing the decrease of antioxidant activities and the increase of hydrogen peroxide. This protection is due to the powerful antioxidant proprieties of GSSE which contains a lot of active compounds such as flavonoids, polyphenols, anthocyanins, proanthocyanidins, procyanidines, and the stilbene derivative resveratrol [8].

In addition, GSSE prevented fatty acids disproportion compared to HFD by lowering levels of oleic, stearic and arachidonic acids into near SD levels. Physiological composition and abundance of fatty acids are critical for lung function. In fact, high levels of oleic, stearic or arachidonic acid can induce the activation of GPR40 or GPR120 leading to an increase of intracellular calcium levels and consequently potentiate acetylcholine-contracted tracheal rings. Moreover, recent studies showed that GSSE improves lung function, increases lung capacity and reduces asthma symptoms [44]. Indeed, GSSE improves lung function, increases lung capacity and reduces asthma symptoms [45]. Indeed, GSSE suppresses inflammation throw the decrease of interleukins levels (IL-3, IL-5 and IL-13) and chemokine (eotaxin-1) [46]. GSSE anti-inflammatory effect is almost similar to that of dexamethasone a powerful synthetic anti-inflammatory drug [45].

In conclusion, HFD induces physio-pathological modification in lung functioning through different processes such inflammation and GRPs activation.

## Conclusions

Here we demonstrate that the asthma and obesity are linked to each other not only through inflammation but also through other physiological parameters such as ions imbalances and oxidative stress. Whereas, GSSE efficiently protected lung from HFD-induced disturbances suggesting its usefulness to protect lung from obesity-induced asthma.

## Abbreviations

CAT: Catalase
FFAs: Free fatty acids
GC–MS: Gaz chromatography-mass spectrometry
GSSE: Grape seed and skin extract
HFD: High fat diet
LDH: Lactate dehydrogenase
MDA: Malondialdehyde
OHS: Obesity-hypoventilation syndrome
POD: Peroxidase
PPAR- γ: Peroxisome proliferator-activated receptor γ
SD: Standard diet
SOD: Superoxide dismutase
